# Supplementary material for: Catching Common Cold Virus with a Net: Pyridostatin Forms Filaments in Tris Buffer That Trap Viruses—A Novel Antiviral Strategy?
Source: Viruses. 2020 Jul 4;12(7):723. doi: 10.3390/v12070723 (PMC7412420; doi:10.3390/v12070723)
Supplement: Supplementary file 1 [file viruses-12-00723-s001.zip › Supplementary File 1_Final.pdf]

Supplementary Material and Methods

Neutrophil isolation

Neutrophils were isolated from peripheral blood of a healthy donor, anticoagulated with EDTA, by using density gradient separation with Ficoll-Paque at 1.073 g/mL (GE Healthcare Bio-Sciences, Sweden) and Histopaque 1.119 g/mL (Sigma-Aldrich, St. Louise, Missouri, USA). Following centrifugation, the neutrophil layer was aspirated, washed with PBS, and the red blood cells were eliminated by hypotonic hemolysis, and isotonicity was then restored with 3% sodium chloride. Isolated neutrophils were resuspended in Hank's Balanced Salt Solution with Ca^{++}/Mg^{++} (HBSS +/+ - Lonza, Walkersville, Maryland, USA), and neutrophil count and purity were determined with the Sysmex XN-350 hemocytometer (Sysmex, Kobe, Japan).

Neutrophil extracellular trap (ET) formation

Neutrophils (2.5×10^5, >90% purity) were seeded on glass coverslips in a 24 well-plate and pre-incubated for 30 min with the neutrophil ET inhibitor Nox2ds-tat (gp91ds-tat, AnaSpec, Fremont, CA, USA) at 10 µM where indicated. Neutrophils were then either left untreated or stimulated with 10 nM phorbol 12 myristate 13-acetate (PMA, Sigma-Aldrich) in a final volume of 500 µL for 2 h at 37°C to allow for neutrophil ET formation. One hundred µL HBSS +/+ without or with RV-A2 (7.2×10^6 TCID50 / well) was then added per well and the plate was incubated on ice for 30 min. Cells were then washed with PBS and fixed with 4% paraformaldehyde for 10 min at room temperature.

ET staining and visualization

For staining of the neutrophil ETs the protocol was adapted from Brinkmann et al. [1]. In short, cells were washed, blocked with 1.5% BSA and 0.1% sodium azide in PBS, and incubated with primary antibodies; rabbit anti-elastase ([EPR7479] Abcam, Cambridge, UK) at 0.13 µg/ml, and mouse anti-RV-A2 monoclonal antibody 8F5 [2] at 2 µg/ml, and detected with Alexa Fluor 488 donkey anti-rabbit (Invitrogen, Waltham, Massachusetts, USA) at 2 µg/ml and IRDye 680RD donkey anti-mouse (LI-COR Biosciences, Lincoln, Nebraska, USA) at 0.5 µg/ml. Hoechst 33342 nucleic acid stain (Invitrogen) was additionally applied at 1:1,000 for 10 min. Coverslips were finally mounted with Fluoromount G (Southern Biotech, Birmingham, Alabama, USA), and samples were viewed with a Zeiss Axio Observer Z1 fluorescence microscope (Zeiss, Oberkochen, Germany) using an EC Plan-Neofluar 100x/1.3 Oil M27 objective or a Zeiss LSM 700 fluorescence confocal microscope using a Plan-Apochromat 63x/1.4 Oil objective.

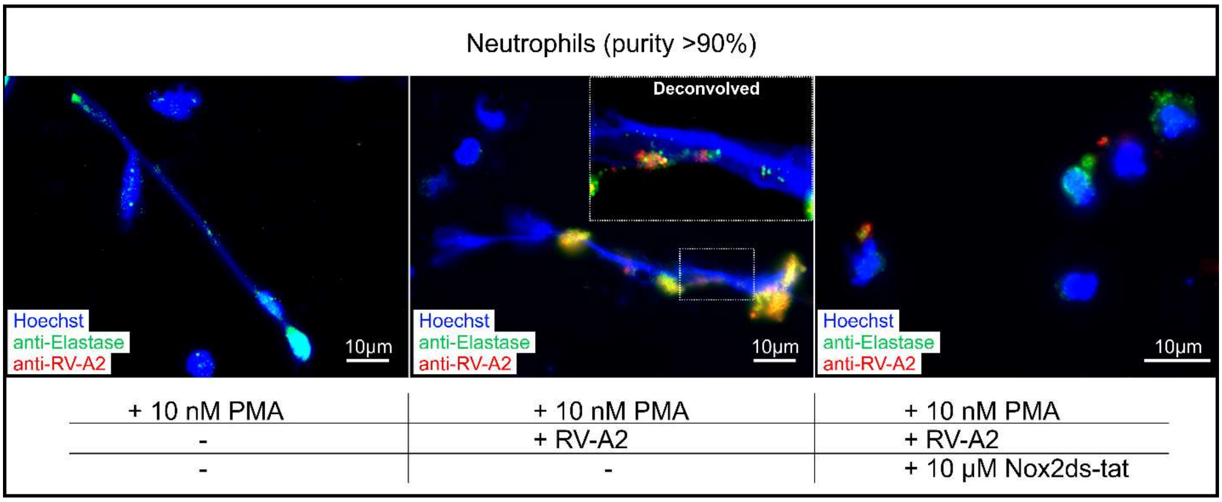

Supplementary Figure S1

Neutrophils were prepared from freshly collected blood from a healthy donor by using centrifugation on a density gradient. They were placed on coverslips and maintained in HBSS +/+ for attachment. Neutrophil ET production was induced via incubation with 10 nM PMA at 37 °C for 2 h in a humid 5% CO_2 atmosphere. The samples were then transferred to 4 °C and incubated with or without RV-A2 (7.2 X 10^6 TCID50 / well) for 30 min. As a negative control, neutrophil ET formation was inhibited with 10 µM Nox2ds-tat. Cells and released material were fixed with 4% formaldehyde in PBS, and blocked with 1.5% BSA and 0.1% sodium azide in PBS. Elastase was immunolabeled with rabbit antibodies against elastase and RV-A2 with the monoclonal antibody 8F5 for 1 h. Samples were washed, incubated with Alexa fluor 488 monkey anti-rabbit antibodies and IRDye 680RD donkey anti-mouse antibodies for 1 h, and finally with 0.1% Hoechst in PBS for 10 min. Cells and released neutrophil ETs were then visualized with the Zeiss Axio Observer Z1 fluorescence microscope using an EC Plan-Neofluar 100x/1.3 Oil M27 objective. The inset shows a deconvolved cutout obtained by using Huygens Professional software version 19.10 (Scientific Volume Imaging, Hilversum, Netherlands)

Supplementary Movie S1

Freshly collected neutrophils from a healthy donor and purified as above by density gradient separation were placed on coverslips for attachment and maintained in HBSS +/+. For induction of neutrophil ET formation, they were incubated for 2 h with 10 nM PMA at 37 °C in a humid 5% CO_2 atmosphere. The neutrophils were then transferred to 4 °C and incubated without or with

RV-A2 (7.2 X 10^6 TCID50 / well) for 30 min. As a negative control, neutrophil ET formation was prevented by the addition of 10 µM Nox2ds-tat. The material was fixed with 4% formaldehyde diluted in PBS and blocked with 1.5% BSA and 0.1% sodium azide in PBS. Further preparation was as above. Cells were then visualized with the LSM 700 fluorescence confocal microscope at a resolution of 0.5 µm on the Z-axis. Fifteen focal planes with a total thickness of 7.5 µm were acquired from a representative cell from the top to the bottom and used for three-dimensional reconstruction with Zen Blue Software (Zeiss). From 60 frames of three-dimensionally reconstructed cells a movie was made at a rate of 5 frames per second; rotation is over the X-axis.

Supplementary reference

1. Brinkmann V, Laube B, Abu Abed U, Goosmann C, Zychlinsky A (2010) Neutrophil extracellular traps: how to generate and visualize them. J Vis Exp (36). doi:10.3791/1724

2. Skern T, Neubauer C, Frasel L, Grundler P, Sommergruber W, Zorn M, Kuechler E, Blaas D (1987) A neutralizing epitope on human rhinovirus type 2 includes amino acid residues between 153 and 164 of virus capsid protein VP2. The Journal of general virology 68 (Pt 2):315-323. doi:10.1099/0022-1317-68-2-315
